# Effects of Konjac Glucomannan on the Gelatinization, Retrogradation, and Dough Properties of Wheat Flour

**DOI:** 10.3390/foods15173054

**Published:** 2026-08-28

**Authors:** Kao Wu, Zixuan Yang, Wenlin Xu, Teng Zhang, Yuxuan Tao, Hong Qian, Qian Zhang, Fatang Jiang

**Affiliations:** 1 Cooperative Innovation Center of Industrial Fermentation (Ministry of Education of Hubei Province), School of Life and Health Sciences, Hubei University of Technology, Wuhan 430068, China; wukao@mail.hbut.edu.cn (K.W.); xuwl336@163.com (W.X.);; 2 School of Nursing and Health Management, Wuhan Donghu University, Wuhan 430212, China; jiangft@mail.hbut.edu.cn; 3 Hubei Key Laboratory of Resource Utilization and Quality Control of Characteristic Crops, College of Life Science and Technology, Hubei Engineering University, Xiaogan 432000, China; zqhbut@163.com; 4 Department of Architecture and Built Environment, Faculty of Engineering, University of Nottingham, Nottingham NG7 2RD, UK

**Keywords:** konjac glucomannan, starch gelatinization, dough properties, textural properties

## Abstract

Konjac glucomannan (KGM), a natural hydrocolloid, is often recognized as an effective texturizing and stabilizing agent in flour-based products. This study systematically investigated the effects of KGM on wheat starch molecules and the gluten protein network. The results showed that different substitution levels (0–5%) of KGM influenced the gelatinization process of starch granules through a thickening effect and competitive hydration. This was specifically manifested as an increase in the pasting viscosity and gelatinization temperature of the system, along with a decrease in gelatinization enthalpy. For textural properties, the KGM addition reduced the hardness of wheat flour gels while enhancing their adhesiveness. Within the dough system, KGM significantly enhanced the gluten network stability, supported by increased water absorption and dough stability time, and decreased degree of softening. Meanwhile, the incorporation of KGM increased the tensile resistance of the dough but decreased the maximum elongation ratio. Furthermore, the storage modulus and loss modulus of the dough showed an increasing trend with higher KGM concentrations. In conclusion, KGM significantly influenced the physicochemical properties and final processing quality of the dough by binding with starch, cross-linking gluten networks, and altering water distribution. This study offered a theoretical foundation for KGM applications in optimizing flour-based product quality.

## 1. Introduction

Wheat starch is the third most produced starch in the world after maize and cassava, and serves as the foundation for the texture of various flour-based products. During dough formation, gluten proteins undergo extensive hydration and form a network via hydrogen bonding and hydrophobic interactions. Starch granules, water, and air are entrapped within this network, and the interactions among these components significantly influence the processing characteristics of the dough [1]. The starch–gluten network is a unique structure of wheat dough [2,3] that imparts good extensibility and gas retention capacity [4].

However, traditional wheat-based products are prone to quality deterioration during storage, such as increased firmness, which is closely dependent on the gelatinization and retrogradation behavior of starch granules. Specifically, starch gelatinization denotes the event where starch chains absorb water and swell under the influence of heat and moisture, leading to the disruption of hydrogen bonds, chain extension, and irreversible collapse of the crystalline structure [5,6]. Retrogradation refers to the recrystallization of gelatinized starch molecules during cooling, during which starch chains and water molecules reassociate and rearrange into a more ordered structure [3,7]. Water migration and molecular rearrangement are key factors influencing the retrogradation process [8]. These changes disrupt the continuity of the gluten network, resulting in product hardening and severely affecting its commercial value and consumer acceptance.

Compared with starch from other sources such as potato and maize, wheat starch has a higher amylose content, resulting in lower pasting parameters [9], which leads to a looser structure and greater tendency to retrogradation in the final products. To improve the processing characteristics of wheat flour, researchers have explored various modification methods. Various modification approaches (e.g., physical, chemical, and enzymatic treatments) have been employed to improve the processing properties of starch-based products. Among these, the addition of hydrocolloids has attracted considerable attention in recent years due to its effectiveness and consumer acceptance [10,11]. Hydrocolloids exhibit good water absorption and thickening properties and are widely used in the improvement of flour-based products [12]. Studies have shown that the introduction of hydrocolloids can interfere with the gelatinization and retrogradation characteristics of wheat starch through mechanisms such as hydrogen bonding, physical entanglement, phase separation, and competitive hydration, as well as by disrupting starch reassociation [13]. Hydrocolloids can also improve the rheological properties of dough and ultimately affect the texture and freeze–thaw stability of products [14,15]. On one hand, it limits the hydration and swelling of starch, thereby delaying gelatinization and inhibiting retrogradation; on the other hand, it enhances the continuity and stability of the gluten network through interactions with gluten proteins.

The effects of different hydrocolloids on starch-based and dough systems varied considerably, depending on their charge characteristics, molecular structure, and concentration [16]. As summarized in a recent review [17], anionic hydrocolloids, such as xanthan gum and sodium alginate, interacted with gluten proteins primarily through electrostatic interactions, significantly enhancing dough stability, tensile resistance, and viscoelastic moduli. In contrast, nonionic hydrocolloids, such as guar gum and konjac glucomannan (KGM), mainly acted through hydrogen bonding and physical entanglement, thereby modulating the structure of flour-based products by improving water binding capacity and dough water absorption, while showing particular advantages in retarding moisture migration and starch retrogradation during storage.

Konjac (*Amorphophallus konjac*) is a perennial monocot herb in the family *Araceae*, and it has a long history of use for food and medicine in East and Southeast Asia [18]. The konjac tuber is rich in active compounds like alkaloids, starch, soluble dietary fiber, and β-carotene, with KGM being the most notable, making up to 60% of the dry matter [19]. Due to its unique physicochemical properties, KGM has attracted considerable research interest in recent decades and is typically sourced from whole konjac tubers [20]. KGM exhibits favorable physicochemical properties such as good thickening, gelling, and water-holding capacities, making it widely used in food processing. KGM can serve as an effective food additive to reduce dough collapse and retard dough retrogradation, thereby improving the quality of flour-based products. Wang et al. [21] found that adding KGM to frozen wheat dough may form a physical barrier on the surface of starch molecules, thereby delaying the retrogradation of frozen wheat flour. Zhao et al. [22] investigated the microstructure and reported that delayed addition of KGM enhanced the cross-linking of gluten proteins, increased the water-holding capacity of the system, and resulted in a softer texture of cooked noodles. Further observations revealed that this treatment led to a highly dense structure in cooked noodles, in which starch molecules were encapsulated. Therefore, KGM acted on both starch and the gluten network by modifying water distribution and through non-covalent interactions such as hydrogen bonding, thereby improving the viscoelastic properties and processing performance of dough.

However, systematic studies on the effects of KGM on the wheat starch–gluten network remained relatively limited, and there was a particular lack of comprehensive analysis regarding the effects of KGM at different addition levels. Therefore, this study aimed to investigate the impacts of different KGM substitution levels (0–5%) on the pasting properties, retrogradation behavior, gel microstructure of wheat flour, as well as the farinograph, textural, and rheological properties of dough. The objective was to interpret the role of KGM in the starch–gluten network from both microstructural and macroscopic perspectives, thereby providing a theoretical basis for expanding the application of KGM in improving the quality of flour-based products.

## 2. Materials and Methods

### 2.1. Materials

Food-grade medium-gluten wheat flour was purchased from Jinshahe Noodle Industry Group Co., Ltd. (Xingtai, China) with a moisture content of 14.3% and a protein content of 11.2%. Premium KGM (*M*_w_ = 9.67 × 10^5^ Da, M/G = 1.6) was purchased from Konson Technology Co., Ltd. (Wuhan, China).

### 2.2. Sample Preparation

Wheat flour was substituted with KGM at levels of 0%, 1%, 2%, 3%, 4%, and 5% (*w*/*w*, based on the total flour blend weight). Each blend was thoroughly mixed to obtain six composite flours with varying KGM substitution levels, and they were coded as K0, K1, K2, K3, K4, and K5, respectively. These samples were sealed and stored for subsequent experiments.

### 2.3. Pasting Properties

Pasting properties for wheat flour and wheat flour/KGM blends were determined utilizing a Rapid Viscosity Analyzer (RVA 4500, Perten Instruments, Hägersten, Sweden) following the method of Rahman et al. [23]. The sample prepared in Section 2.2 was accurately weighed (3.0 g) and then mixed with 25 mL of 0.06 mol/L AgNO_3_ solution in an RVA aluminum canister. The addition of AgNO_3_ in the RVA analysis was intended to inhibit endogenous α-amylase activity present in wheat flour [24]. Then the samples were held at 50 °C for 1 min, and the heating rate was set at 12 °C/min up to 95 °C, held at 95 °C for 2.5 min. Subsequently, they were cooled to 50 °C at the same rate and kept there for 2 min. The stirring speed was maintained at 960 r/min for the initial 10 s to homogenize the dispersion and was kept at 160 r/min until the end of the measurement. The following pasting parameters were determined from the RVA profile: peak viscosity is the maximum viscosity obtained during heating; trough viscosity is the minimum paste viscosity obtained during the holding period; final viscosity is the viscosity measured at the end of the cooling process; breakdown viscosity represents the differences between peak viscosity and trough viscosity; and setback viscosity represents the differences between trough viscosity and final viscosity.

### 2.4. Gel Microstructure

The microstructure of the gels was observed using the method from Gong et al. [25]. After the RVA test, the gel samples were refrigerated at −18 °C for 24 h and freeze-dried. Then, they were attached to a stub coated with conductive adhesive and treated with vacuum gold spraying under an average current of 15 mA and a vacuum degree of 7–8 Pa. At an acceleration voltage of 5 kV, the samples were observed at a magnification of ×200 by using an SEM (JSM6390LV, JEOL, Tokyo, Japan).

### 2.5. Texture Profile Analysis (TPA)

The textural characteristics of wheat flour gel were measured following the procedure of Dangi et al. [26] with some modifications. The gel samples collected from the RVA test were covered with plastic wrap and stored at 4 °C for 24 h or 72 h. The textural properties of the gels were measured on a TA-XT Plus texture analyzer (Stable Micro Systems, Surrey, UK) with the P/50 probe under the TPA mode. The speeds for pre-test, test, and post-test were 2.0 mm/s, 1.0 mm/s, and 2.0 mm/s, respectively. Compression strain was fixed at 50% with a time gap of 5 s between two compression cycles. The trigger force was maintained at 5.0 g. Hardness (the peak force during the first compression), springiness (the ratio of the compression time of the second compression to that of the first compression), and adhesiveness (energy required to withdraw the probe after a single compression) were recorded.

### 2.6. Thermal Properties

Thermal properties were measured by differential scanning calorimetry (DSC1, Mettler-Toledo, Greifensee, Switzerland) using the method from Wang et al. [27]. The sample (2 mg) was mixed with 20 mg of deionized water before being hermetically sealed in a DSC pan and equilibrated at 4 °C for 12 h. An empty DSC pan served as the reference. The temperature range was 25–95 °C with a heating rate of 10 °C/min. The onset temperature (T_O_), peak temperature (T_P_), conclusion temperature (T_C_), and the enthalpy of gelatinization (ΔH) were recorded.

### 2.7. Farinographic Properties

The farinographic properties of the blends (prepared in Section 2.2) were measured using a farinograph (JFZD-300, Fangce Precision Instrument Technology Co., Ltd., Shenzhen, China) according to the method from Sim et al. [28]. The obtained farinogram provided information on Farinograph Quality Number (FQN, the time from the start of the test to a 30 BU drop from the peak center), dough development time (the time to reach maximum consistency), stability time (the time that the curve remains above 500 BU), degree of softening (the consistency difference between the peak time point and 12 min after peak time is reached) and water absorption values (the amount of water required to achieve a dough consistency of 500 BU).

### 2.8. Dough Preparation

Dough was prepared using a slightly modified method from Liu et al. [29]. According to the optimal water absorption determined by the farinograph, 90% of the corresponding deionized water was calculated, weighed, and mixed into the KGM–wheat flour mixture (300 g) prepared in Section 2.2. The mixture was kneaded in a dough mixer at low speed for 3 min, followed by medium speed for 5 min. The kneaded dough was then placed into a square mold, covered with plastic wrap, and allowed to rest at room temperature for 10 min prior to analysis.

### 2.9. Textural Properties of the Dough

Both the compression and tensile properties of dough were determined using the same textural analyzer but with different probes. The former properties were tested according to Su et al. [30]. Specifically, 50 g of the dough prepared in Section 2.8 was shaped into a sphere (5 cm in diameter) and sealed with plastic film to avoid evaporation. TPA was performed using a TX.XT plus texture analyzer (Stable Micro Systems, Surrey, UK) with the P/36R probe. The test parameters were set similar to those described in Section 2.5, except that the post-test speed was set to 1 mm/s. All other parameters remained unchanged.

A dough (5 g) prepared in Section 2.8 was sheeted and cut into uniform strips (5 cm in length, 2 cm in width, and approximately 3 mm in thickness). Tensile tests were measured with the A/KIE probe at a stretching distance of 20 mm. The pre-test, test, and post-test speeds were set at 2.0 mm/s, 4.0 mm/s, and 10.0 mm/s, respectively. Tensile resistance (N, the maximum tensile force at fracture) and maximum elongation ratio (%, the ratio of the extended length to the original length) were recorded.

### 2.10. Rheological Properties

Rheological characteristics of KGM–wheat dough were determined using a rheometer (MCR 92, Anton Paar, Graz, Australia) following the method from Zhang et al. [31] with minor modifications. The dough (5 g) prepared in Section 2.8 was placed in a parallel plate geometry (40 mm diameter and 2 mm gap) for frequency scanning tests. Excess material around the edges was wiped off slightly with a rubber scraper, and Vaseline was applied to the plate edges to prevent evaporation during the experiment. Frequency sweep tests were performed at 25 °C after a 300 s equilibration, using a constant strain amplitude of 1.0% over a frequency range of 0.1–10 Hz. The storage modulus (G′) and loss modulus (G″) were recorded throughout the measurement.

### 2.11. Statistical Analysis

Each experiment was conducted in triplicate. Statistical evaluations were conducted using Origin 2018 (Origin Lab Corporation, Northampton, MA, USA) and SPSS Statistics 27 (IBM SPSS, Chicago, IL, USA). One-way analysis of variance (ANOVA) followed by Tukey’s post hoc test was applied for group comparisons, with statistical significance set at *p* < 0.05. Results were presented as mean ± standard deviation (SD).

## 3. Results and Discussion

### 3.1. Effect of KGM on the Pasting and Thermal Properties of Wheat Flour

#### 3.1.1. Pasting Properties

The pasting process is a critical determinant of starch-based product quality [32]. The pasting parameters of wheat flour with different KGM substitutions are presented in Table 1. The peak, trough, and final viscosities, as well as the breakdown and setback of wheat flour, all increased progressively with the substitution of KGM. The increase in peak viscosity values was partly due to KGM forming a colloidal solution upon addition, and partly attributable to interactions between polysaccharides and starch molecules [33]. During starch granule swelling, friction and interactions between starch granules and KGM chains, as well as between starch granules themselves, reached their maximum. Consequently, KGM exhibited a synergistic thickening effect.

Compared to peak viscosity, trough viscosity decreased by nearly half across all groups, reflecting the rupture and disintegration of swollen starch granules. However, as the KGM substitution increased, trough viscosity also rose gradually. This resilience stemmed from the long molecular chains of KGM, which sustained high viscosity post-shear disruption via physical entanglement, hydrogen bonding, and steric hindrance with starch molecules in the blend (especially solubilized amylose chains). Furthermore, the complete unfolding of KGM molecular chains required sufficient hydration time [34]. Therefore, they only underwent partial swelling during the RVA test, which increased viscosity.

Setback viscosity measures the ability of starch molecules to reassociate amylose chains after high-temperature gelatinization, indicating retrogradation tendency. Generally, the setback viscosity correlates with starch recrystallization, and a higher value indicates faster short-term retrogradation. However, in the present KGM–wheat mixed system, the elevated setback viscosity in RVA should not be directly equated with aggravated starch retrogradation. During the cooling phase, the significant increase in final and setback viscosities was more likely to stem from the viscosity contribution of KGM. As the temperature decreased, the hydrogen bond network of KGM rapidly rebuilt, causing a sharp rise in viscosity [35], which directly contributed to the viscosity of the KGM–wheat system.

Breakdown represents the resistance of the swollen starch system to thermal and shear forces. A higher breakdown indicates greater instability in the starch granule structure. Some studies have suggested that the increase in breakdown resulted from heightened shear stress at a constant shear rate due to elevated system viscosity, thereby compromising the integrity of starch granules [36]. Alternatively, during the high-temperature stage, KGM exhibited intensified molecular chain motion that weakens hydrogen bonding interactions, leading to a significant decrease in trough viscosity. Macroscopically, this manifested as an increase in breakdown with rising KGM content.

#### 3.1.2. Thermal Properties

Gelatinization data for wheat flour with KGM substitution are summarized in Table 2. Compared to the control group, all KGM-substituted groups exhibited increased T_O_, T_P_, and T_C_ values, while gelatinization enthalpy decreased with increasing KGM dosage. For T_O_ and T_P_, no significant differences were observed among samples at higher KGM substitutions, indicating that KGM concentration had a limited effect on the initial dissociation of starch crystalline structure during gelatinization. Previous research [37] suggested that non-starch polysaccharide (NSP) addition significantly affects T_C_. This occurred because NSPs phase-separate from leached amylose chains, creating locally enriched NSP regions [38]. Such areas inhibited water penetration into the most stable crystalline domains, suppressing hydration within starch crystals. Crucially, this process disrupted the dissociation of amylopectin double helices, supporting our findings. The enthalpy of gelatinization (∆H) represents the energy required to disrupt the starch double helices, determined by the crystalline order of the starch granule and the organization density of the double helices. As shown in the table, increasing KGM substitution gradually reduced the gelatinization enthalpy of the system, likely owing to the diluting effect on starch components. Furthermore, Liu et al. [39] observed a similar phenomenon when studying the effect of Malva crenata polysaccharide on wheat starch gelatinization characteristics. The authors attributed this phenomenon to the strong water absorption of Malva crenata polysaccharide, inhibiting the diffusion rate of water into the crystalline regions of starch, thereby delaying the gelatinization process and limiting the full gelatinization of starch granules. However, other studies indicated that the effect of KGM on starch molecule gelatinization enthalpy was not constant, depending on the content and structural type of crystalline regions within the starch molecule [40]. KGM substitution altered interactions between starch and water molecules, ultimately influencing parameters such as starch viscosity and gelatinization temperature.

### 3.2. Effect of KGM on the Microstructural and Textural Properties of Wheat Flour Gel

#### 3.2.1. Microstructure of the Gels After Lyophilization

**Figure 1:**
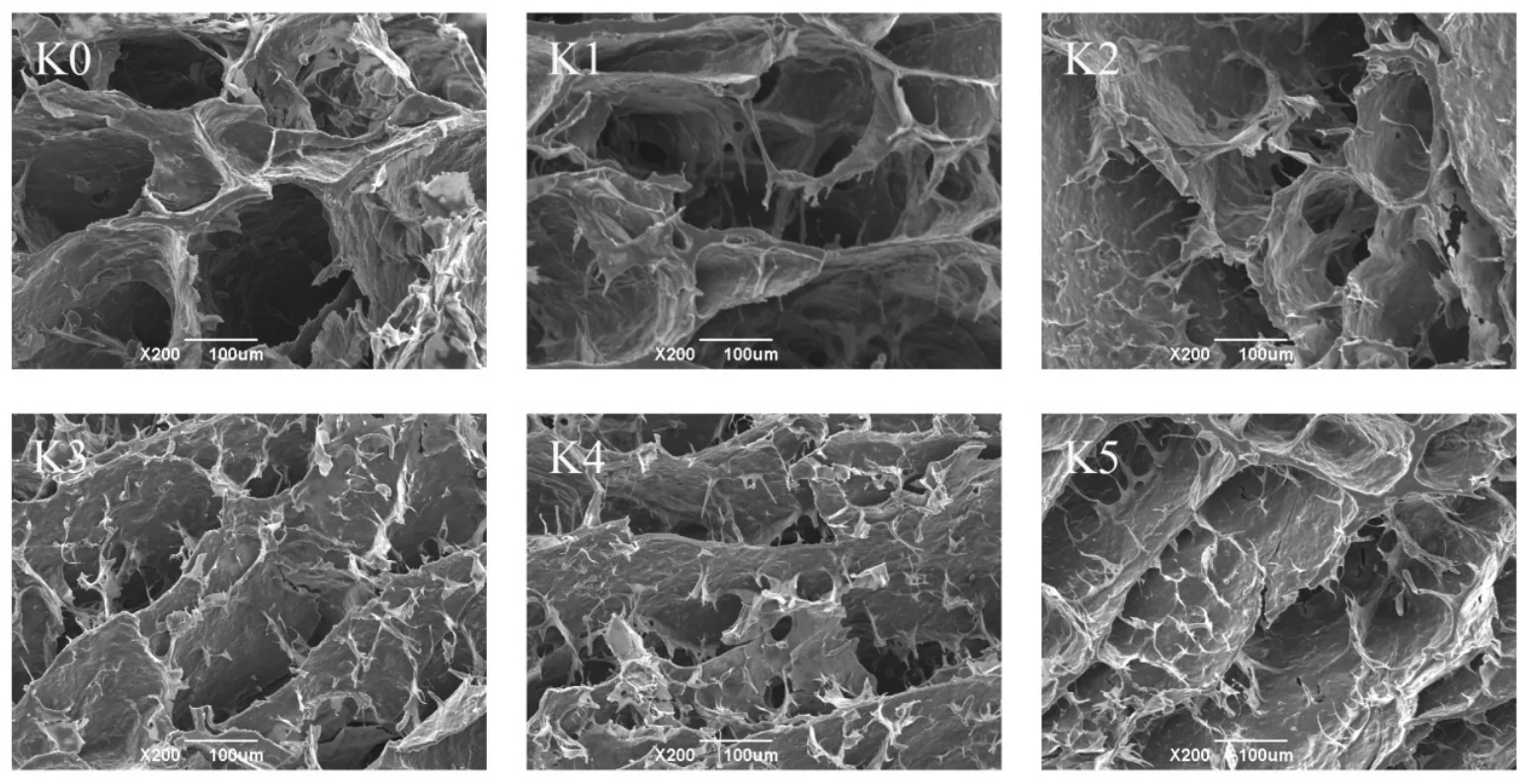
Microstructure of lyophilized wheat flour gel with different substitution levels of KGM.

The gel microstructures of the wheat flour–KGM mixtures at varying KGM substitution levels are shown in Figure 1. Before observation, all samples were subjected to a freeze-drying treatment. Although this altered the gel microstructure due to ice crystal growth and sublimation, the observed structural differences among the samples were still meaningful as indirect evidence. A loose structure and large pores were observed in the wheat flour gel of the control group (K0). The porous structure was caused by the sublimation of ice crystals, leaving behind irregular cavities and channels. During starch gelatinization, water molecules and starch molecules formed a new gel network structure via hydrogen bonding. The rearrangement of amylose chains released during gelatinization redistributed water, forming large, irregular ice crystals under freezing conditions. This led to uneven porosity distribution after freeze-drying [41].

As depicted in SEM, the addition of KGM affected the pore size of the flour gel. As KGM content increased, hydrogen bonding interactions and physical entanglement between KGM and starch molecules occurred, reducing rearrangement and aggregation during starch retrogradation. Concurrently, the thickening action of KGM and its ability to fill pores within the gel network collectively reduced pore size in the wheat flour gel, resulting in a denser and more regular structure [42].

#### 3.2.2. Textural Properties of KGM/Wheat Flour Gels

**Figure 2:**
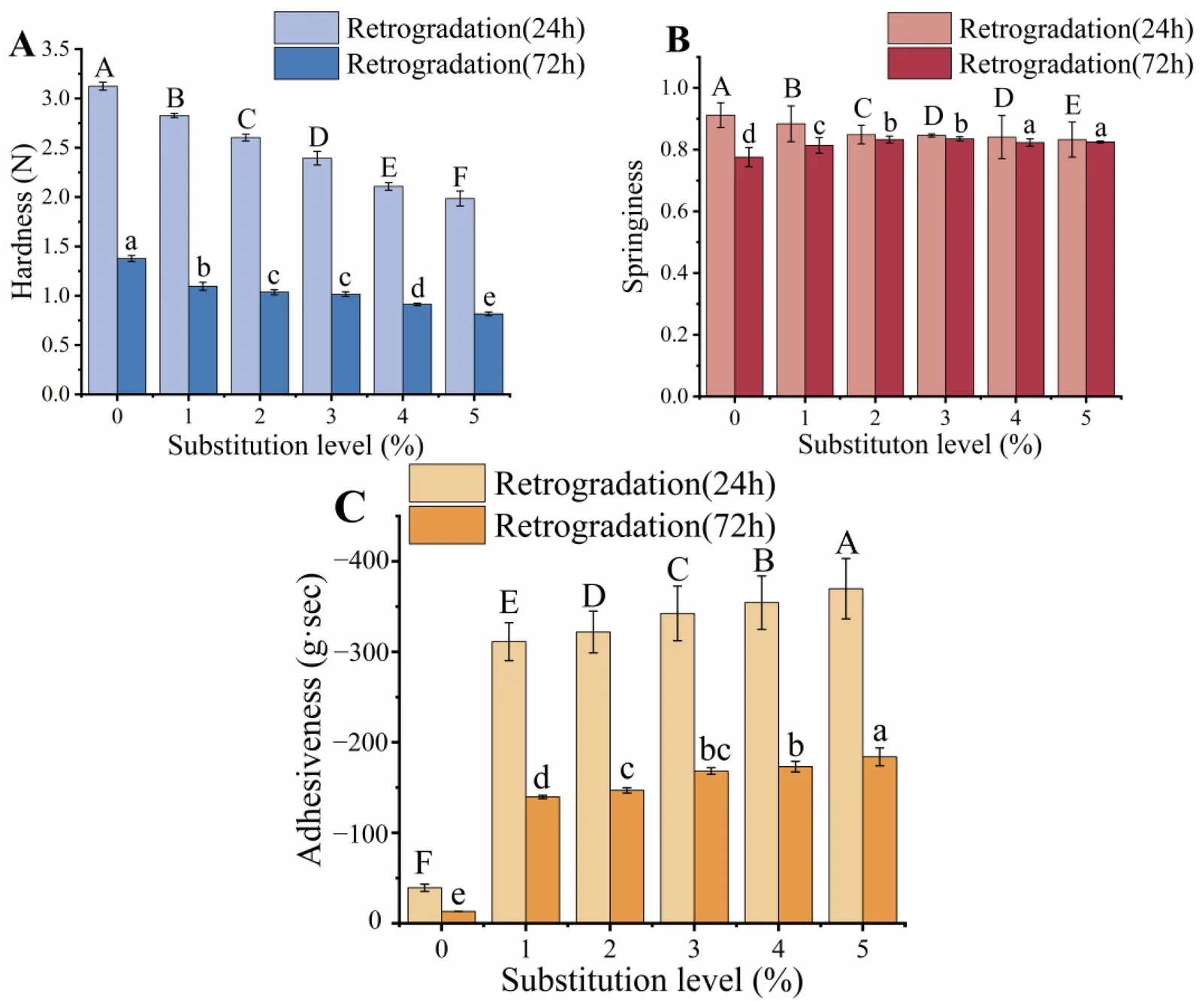
Effects of different KGM substitution levels on the hardness (**A**), springiness (**B**), and adhesiveness (**C**) of wheat flour after retrogradation. Different uppercase letters reveal significant differences among the data at 24 h of storage (*p* < 0.05); different lowercase letters indicate significant differences among the data at 72 h of storage (*p* < 0.05).

Effects of different KGM substitution levels on the hardness, springiness, and adhesiveness of wheat flour during retrogradation are shown in Figure 2A–C. The data indicated that the hardness of all gels decreased with extended storage time, which was related to water redistribution within the flour gel network. Notably, within the same observation period, the hardness of wheat flour gels decreased with increasing KGM substitution levels, as this trend was driven by the molecular interactions between starch and the added polysaccharide [43]. The long chains of KGM retarded the starch recrystallization process through steric hindrance effects [44], thereby inhibiting starch retrogradation and avoiding the shrinkage of the flour gel. Additionally, the strong hydrophilicity of KGM enhanced water-holding gel capacity. The decrease in the hardness of the gel was influenced collectively by these factors. In the control group, lower hardness was observed at 72 h than at 24 h, indicating inhibited starch retrogradation. It was demonstrated by Rahman et al. [23] that AgNO_3_ significantly reduced the final viscosity and setback value of rice flour. Another study [45] reported that NO_3_^−^ ions could inhibit starch retrogradation and thus a weaker three-dimensional network of starch gel was formed, supported by the reduced gel strength during cold storage. Therefore, this may be attributed to the impact of AgNO_3_ on the gel structure.

No significant differences in springiness were observed among the different KGM addition groups at either 24 h or 72 h of storage in Figure 2B. This result indicated that the substitution of KGM had little effect on the ability to recover shape after deformation of wheat flour gel. This phenomenon occurred because the addition of KGM helped maintain the structural continuity of the gel network during the retrogradation of starch. A similar phenomenon was reported by Liu et al. [46], who found that the addition of Creeping fig seed polysaccharide had no significant impact on the springiness of potato starch gels, and it was explained by the combined impacts of Creeping fig seed polysaccharide-starch association and water migration. As shown in Figure 2C, the adhesiveness of starch samples containing KGM was significantly higher than that of wheat starch samples without KGM, which can be attributed to the excellent thickening ability of KGM. The adhesiveness of gel samples gradually increased with KGM substitution after 24 h of retrogradation. Furthermore, the adhesiveness of wheat flour gel samples after 72 h of retrogradation decreased compared to that after 24 h of retrogradation. This phenomenon can be attributed to the thickening effect of KGM macromolecules, which restricted the mobility of starch chains and inhibited retrogradation. This interpretation was consistent with the observed changes in gelatinization parameters.

### 3.3. Effect of KGM on Farinograph Properties of Wheat Flour

Parameters derived from farinograph curves serve as essential tools for evaluating flour quality, as they reflect the fundamental rheological properties of flour. Table 3 shows the effects of different KGM substitution levels on the farinograph properties of wheat flour. KGM substitution markedly influenced farinograph properties, with wheat dough water absorption, dough stability time, and development time all enhanced significantly as KGM substitution levels rose, contrasting with findings by Sim, Noor Aziah and Cheng [28], who observed that adding 0.8% KGM to wheat flour shortened dough stability time. This discrepancy likely stemmed from differing protein levels in the flour samples. In the present study, the dough water absorption rate increased from approximately 65% to 110% as the KGM addition level increased. This wide range of variation was highly correlated with the water absorption preferences of different flour-based products. It was reported that although the water absorption of whole wheat flour with fine bran (approximately 63%) was higher than that with coarse bran, the noodles made from the former exhibited better smoothness and appearance [47]. It was also found that when the addition level of KGM reached 1.5%, the water absorption of the dough increased to 75%, and the steamed bread prepared with this formulation received a relatively high sensory score [13]. Presently, utilizing flour with a water absorption exceeding 100% in dough-based products is rare, as this requires the addition of large amounts of hydrocolloids and might lead to different processing properties of the final product quality.

The dough development time of wheat flour increased with higher KGM concentrations, suggesting that KGM prolonged the time required for the system to reach optimal hydration by competing for water with gluten and starch. Enhanced dough water absorption improved yield in baked goods and mitigated surface cracking of dough. This aligns with prior research showing that adding other polysaccharides, such as cassava polysaccharides (NSP), significantly increased wheat dough water absorption while markedly reducing weakening [48]. The extensive hydrogen bond networks between the -OH groups in polysaccharide molecules and water account for this phenomenon.

With the degree of softening and dough stability values indicating the dough tolerance and breakdown rate, a decline in the degree of softening and an elevation in stability both signified stronger dough strength and greater resistance to breakdown [49]. During the initial stages of dough formation, KGM competitively bound a large amount of free water, thereby delaying the full hydration and unfolding of gluten proteins and prolonging dough formation time. As kneading progressed, KGM molecular chains enhanced the continuity of the starch–gluten protein structure through hydrogen bonding and physical entanglement [50], making it more resistant to mechanical shear and significantly improving dough stability. Liu et al. [51] reported similar findings, concluding that dough formation time positively correlates with gluten strength and that polysaccharide addition positively impacts dough stability.

### 3.4. Effect of KGM on the Mechanical Properties of Wheat Dough

#### 3.4.1. Textural Properties

**Figure 3:**
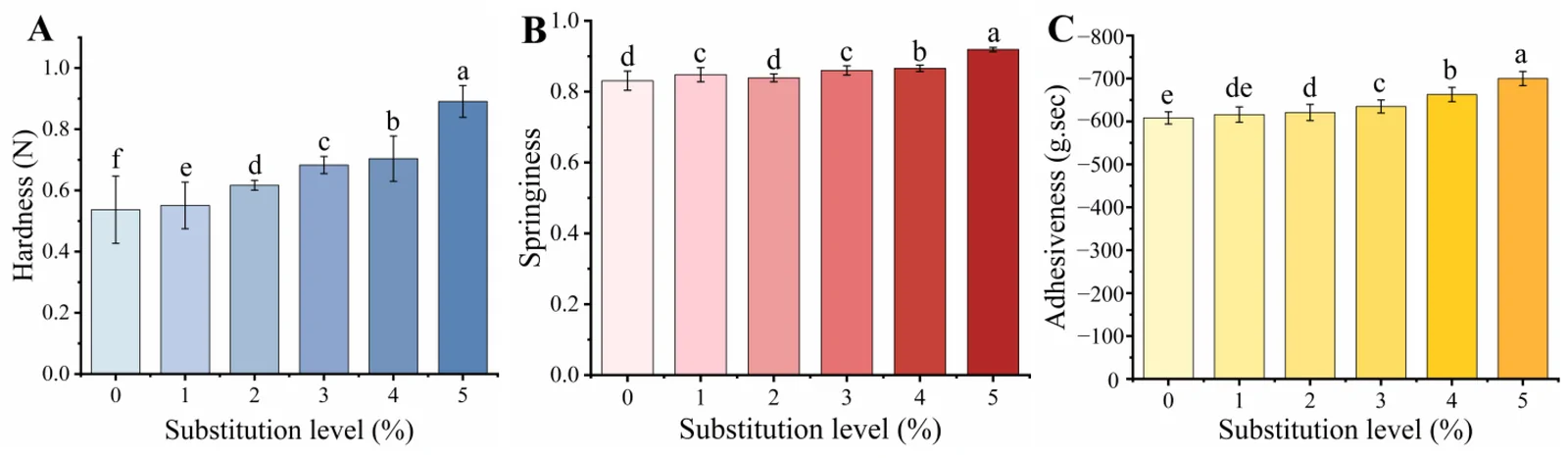
Effects of different KGM substitution levels on the hardness (**A**), springiness (**B**), and adhesiveness (**C**) of wheat dough. Different lowercase letters indicate significant differences among the data (*p* < 0.05).

As shown in Figure 3A–C, the addition of KGM significantly increased the hardness and adhesiveness of the dough. The starch–gluten protein network constituted the unique structure of wheat dough, conferring extensibility and elasticity. With increasing KGM substitution, the hardness, springiness, and adhesiveness of KGM–wheat dough progressively increased. Analysis of the results from the farinograph properties of wheat flour indicated that dough formation primarily involved the cross-linking of gluten proteins, starch granules, and water to form a three-dimensional spatial network structure. The addition of NSP affected this process. KGM substitution reduced the degree of softening and enhanced stability, strengthening the starch–gluten protein network. Concurrently, long chains of KGM formed gel–protein structures with the gluten network involving non-covalent forces such as hydrogen bonds [30], thereby increasing the dough’s hardness. The slight increasing trend in springiness can be attributed to KGM not only filling the voids between starch granules and gluten proteins but also promoting physical bonding between gluten proteins and starch [25]. Dough water retention capacity and surface wettability were significantly increased by the strong hydrophilicity of KGM. Simultaneously, the viscous system formed by KGM molecules adsorbing water enhances separation resistance at the dough–probe interface, thereby improving the adhesiveness of the dough system. Studies showed that moderate elasticity and viscosity of dough were essential for gas retention during proofing [52], and the addition of KGM had been found to improve dough gas-holding capacity. Furthermore, the enhanced water absorption capacity of hydrocolloids reduced the dehydration rate of bread [53], which positively affected the quality of fermented flour products such as bread and also improved the quality of frozen dough.

#### 3.4.2. Tensile Properties

**Figure 4:**
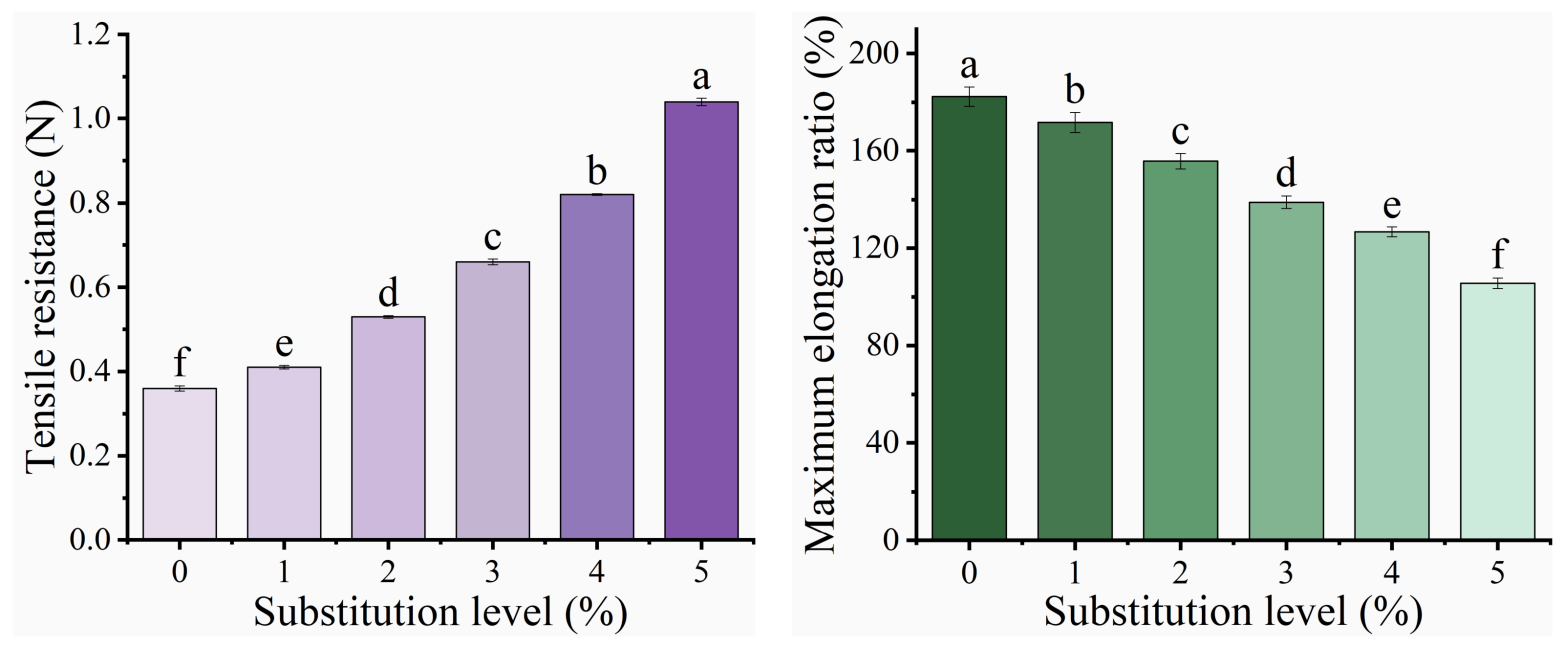
Tensile properties of wheat dough with different substitution levels of KGM. Different lowercase letters indicate significant differences among the data (*p* < 0.05).

As the KGM content increased, the tensile resistance of wheat dough significantly rose, while the maximum elongation rate correspondingly decreased (Figure 4). This can be attributed to the tightly bound gel–protein structure, which enhanced dough gluten strength to a certain extent. Research indicated that the branching rate and continuity of gluten proteins significantly influenced dough extensibility [54]. Under equivalent stress conditions, the incorporation of non-gluten components (KGM) disrupted the structural integrity of the gluten protein network. KGM was tightly entangled with gluten proteins, restricting the slip of gluten protein molecular chains. This led to earlier dough rupture during stretching, thereby reducing the extensibility distance. Sun, et al. [4] observed in whole wheat dough studies that non-gluten components (wheat bran) disrupted the continuous cross-linking of gluten proteins, thereby reducing dough extensibility.

#### 3.4.3. Rheological Properties

**Figure 5:**
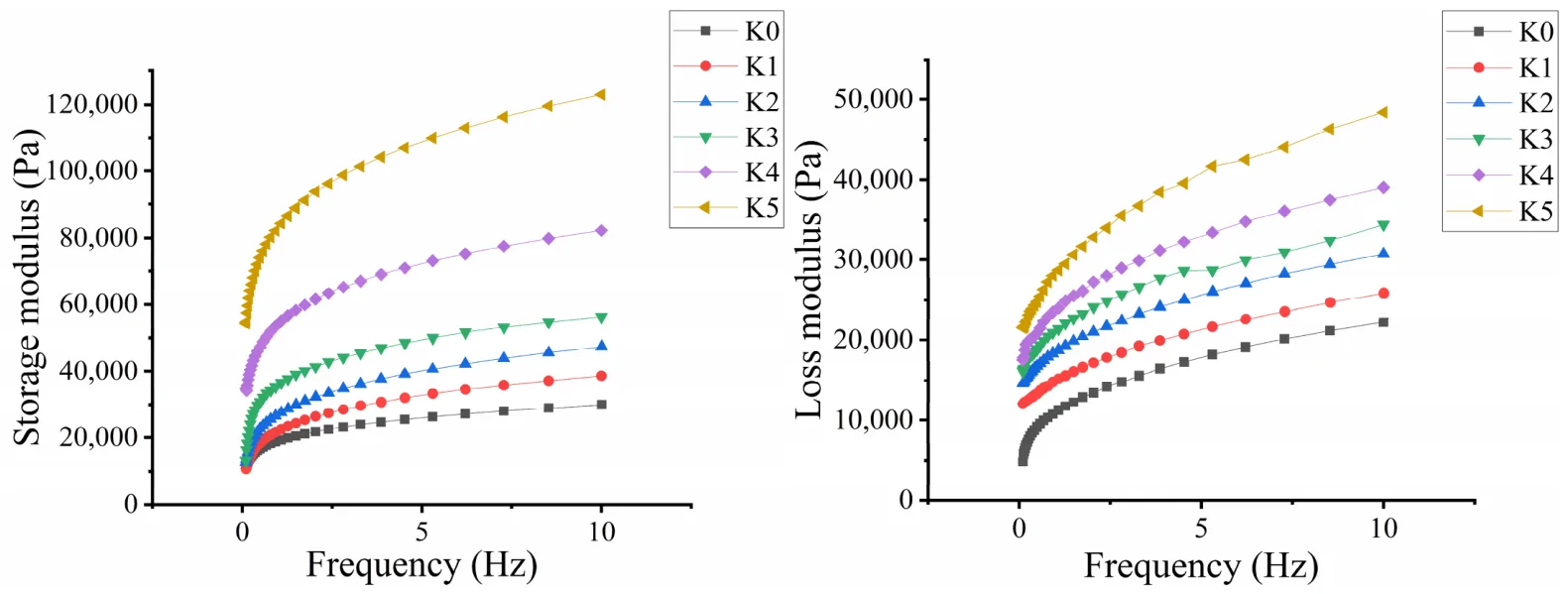
Storage modulus (G′) and loss modulus (G″) of wheat dough with different KGM substitution levels.

The rheological characteristics of dough determined its processing performance and influenced the quality of the final product. The storage modulus (G′) and loss modulus (G″) distinguished the viscoelasticity of the test material, with higher G′ indicating stronger elasticity and higher G″ reflecting greater viscosity. All dough samples were prepared using 90% of the corresponding farinograph water absorption value to facilitate dough handling, and the effect of different KGM substitution levels on the rheological properties of wheat dough is shown in Figure 5. As the scanning frequency increased, the elastic modulus consistently exceeded the viscous modulus, suggesting greater elasticity than viscosity and exhibiting solid-like behavior. Furthermore, no crossover between G′ and G″ values occurred, indicating no phase transition and a weak gel state.

Compared to the control group, dough samples with KGM substitutions exhibited higher elasticity and viscosity values. Both G′ and G″ values increased significantly with rising KGM levels. Dough with higher free water content demonstrated lower stability [55]. The higher storage modulus (G′) and loss modulus (G″) observed in KGM-containing doughs were partially attributed to the structural features of the KGM used in this study. Previous studies showed that KGM with higher mannose content exhibited enhanced chain rigidity and stronger intermolecular entanglements, which contributed to higher solution viscosity [56]. Therefore, the relatively high M/G ratio (1.6:1) of the KGM in this study could facilitate stronger hydrogen bonding and physical entanglement between KGM and gluten proteins, thereby reinforcing the gluten network and ultimately resulting in the increased G′ and G″ values.

As KGM content increased, the water absorption of wheat dough rose. KGM molecules altered the binding mode between gluten proteins and water molecules, reducing free water content in the system and strengthening the gluten network. Simultaneously, KGM chains formed physical entanglements or hydrogen bonds with gluten proteins, further enhancing the elasticity and viscosity of the network. The change in G′ value after KGM substitution to wheat dough was more pronounced than the change in G″ value, indicating that KGM exerts a more significant effect on the elastic properties of wheat flour gel than on its viscous properties. It should be noted that the 5% substitution level examined in this study was higher than the concentrations typically used in conventional bakery applications. Although this level may not be directly applicable to standard bread production, it was selected as an exploratory upper bound to facilitate the observation of threshold effects in structural and rheological changes. For practical applications, lower concentrations within the typical industrial range are considered more feasible and warrant further product-specific evaluation.

## 4. Conclusions

This study investigated the effects of KGM on the gelatinization and retrogradation properties of wheat starch, as well as the structural characteristics of the gluten network. The results demonstrated that KGM significantly influenced the processing properties and final quality of dough. Specifically, the substitution of KGM increased the pasting viscosity and gelatinization temperature of wheat starch, while reducing the gelatinization enthalpy. This phenomenon was mainly attributed to the competition for free water between KGM and starch molecules, which altered the hydration environment of starch granules. Further analysis of farinograph properties revealed that KGM significantly enhanced the water absorption, dough development time, and dough stability, which were closely related to its excellent hydrophilicity and water-holding capacity. Within the dough system, the observed rheological and textural improvements were speculated to arise from non-covalent interactions (e.g., hydrogen bonding and physical entanglement) between KGM long chains and the leached amylose/gluten network. These interactions reinforced the stability of the gluten network, thereby improving the mechanical strength, tensile resistance, and storage modulus (G′) of the dough, conferring enhanced processing tolerance.

Overall, the physicochemical properties of both starch and dough showed a strong correlation with the KGM content. From a systematic evaluation of dough rheological, tensile, and textural properties, a KGM substitution level of 3% was recommended for most fermented flour products, as it provided a reasonable balance between dough strengthening and extensibility retention. Based on comprehensive improvements in pasting, textural, and rheological properties, we proposed that KGM-supplemented wheat flour was suitable for fermented flour products such as bread, steamed bread, noodles, and frozen dough-based products by enhancing water absorption, dough handling, and anti-retrogradation during storage. This study offers a theoretical reference for the application in the quality improvement of flour products. However, the molecular-level binding behavior between KGM and gluten proteins, particularly their effects on protein secondary structure and disulfide bond formation, warrants further in-depth investigation. Future work can be conducted toward the evaluation of flour-based bakery food, such as baking performance, staling kinetics over a longer period, and sensory acceptability, to further verify the practical applicability of KGM addition.

## Figures and Tables

**Table 1:** Effect of different KGM substitution levels on the pasting properties of wheat flour.

KGM Substitution (%)	Peak Viscosity (cP)	Trough Viscosity (cP)	Final Viscosity (cP)	Breakdown Viscosity (cP)	Setback Viscosity (cP)
0	2155.7 ^e^ ± 4.2	1380.3 ^e^ ± 3.7	2193.0 ^f^ ± 4.5	735.3 ^e^ ± 1.3	812.7 ^f^ ± 1.1
1	2975.3 ^d^ ± 5.4	1553.3 ^d^ ± 1.6	2411.3 ^e^ ± 11.2	1422.0 ^d^ ± 4.2	858.0 ^e^ ± 1.9
2	3607.7 ^c^ ± 2.3	1685.7 ^c^ ± 6.5	2645.3 ^d^ ± 13.0	1922.3 ^c^ ± 3.1	959.7 ^d^ ± 2.5
3	3763.3 ^c^ ± 1.3	1798.3 ^b^ ± 9.6	2864.3 ^c^ ± 14.3	1965.7 ^c^ ± 4.4	1066.0 ^c^ ± 3.2
4	4540.0 ^b^ ± 4.5	1939.3 ^a^ ± 11.3	2981.3 ^b^ ± 11.1	2600.7 ^b^ ± 5.8	1042.0 ^b^ ± 2.4
5	5114.5 ^a^ ± 1.3	2033.5 ^a^ ± 16.2	3201.0 ^a^ ± 1.9	3081.0 ^a^ ± 8.6	1167.5 ^a^ ± 3.5

All values are expressed as mean ± standard deviation (*n* = 3). Means in the same column with different superscript letters differ significantly (*p* < 0.05).

**Table 2:** Effect of different KGM substitution levels on the thermal properties of wheat flour.

KGM Substitution (%)	T_O_ (°C)	T_P_ (°C)	T_C_ (°C)	ΔH (J/g)
0	55.9 ^d^ ± 0.1	61.5 ^f^ ± 0.0	66.9 ^a^ ± 0.1	2.9 ^a^ ± 0.0
1	56.0 ^d^ ± 0.0	62.8 ^e^ ± 0.0	67.3 ^b^ ± 0.0	2.7 ^b^ ± 0.0
2	56.1 ^d^ ± 0.1	63.1 ^d^ ± 0.0	68.0 ^c^ ± 0.0	2.3 ^c^ ± 0.0
3	56.7 ^c^ ± 0.1	63.8 ^c^ ± 0.0	68.4 ^d^ ± 0.0	2.1 ^d^ ± 0.0
4	58.4 ^b^ ± 0.1	64.6 ^b^ ± 0.0	69.1 ^e^ ± 0.0	1.9 ^e^ ± 0.0
5	59.5 ^a^ ± 0.1	65.8 ^a^ ± 0.0	70.2 ^f^ ± 0.0	1.5 ^f^ ± 0.0

All values are expressed as mean ± standard deviation (*n* = 3). Means in the same column with different superscript letters differ significantly (*p* < 0.05).

**Table 3:** Farinograph properties of wheat flour at different KGM substitution levels.

KGM Substitution (%)	Water Absorption (%)	Dough Development Time (min)	Farinograph Quality Number (mm)	Degree of Softening (BU)	Dough Stability (min)
0	65.1 ^f^ ± 1.4	4.7 ^f^ ± 0.1	81 ^f^ ± 6.2	48 ^a^ ± 2.4	6 ^f^ ± 0.2
1	72.6 ^e^ ± 2.1	5.4 ^e^ ± 0.4	86 ^e^ ± 2.4	42 ^b^ ± 2.1	6.7 ^e^ ± 0.2
2	85.4 ^d^ ± 1.5	6 ^d^ ± 0.2	91 ^d^ ± 8.1	36 ^c^ ± 3.3	7.2 ^d^ ± 0.2
3	95.1 ^c^ ± 1.7	6.5 ^c^ ± 0.5	99 ^c^ ± 5.3	35 ^d^ ± 2.4	8.5 ^c^ ± 0.2
4	104.1 ^b^ ± 2.3	10.3 ^b^ ± 0.3	103 ^b^ ± 2.1	33 ^e^ ± 1.9	11.5 ^b^ ± 0.4
5	110.1 ^a^ ± 3.1	12.5 ^a^ ± 0.5	115 ^a^ ± 2.0	31 ^f^ ± 1.7	20.8 ^a^ ± 0.3

All values are expressed as mean ± standard deviation (*n* = 3). Means in the same column with different superscript letters differ significantly (*p* < 0.05).

## Data Availability

The original contributions presented in this study are included in the article. Further inquiries can be directed to the corresponding author.

## Abbreviations

The following abbreviations are used in this manuscript:

KGM	Konjac glucomannan
T_O_	Onset temperature
T_P_	Peak temperature
T_C_	Conclusion temperature
ΔH	enthalpy of gelatinization
NSP	non-starch polysaccharide
G′	storage modulus
G″	loss modulus
